# MRPL15 is a novel prognostic biomarker and therapeutic target for epithelial ovarian cancer

**DOI:** 10.1002/cam4.3907

**Published:** 2021-05-02

**Authors:** Haoya Xu, Ruoyao Zou, Feifei Li, Jiyu Liu, Nannan Luan, Shengke Wang, Liancheng Zhu

**Affiliations:** ^1^ Department of Obstetrics and Gynecology Shengjing Hospital of China Medical University Shenyang China; ^2^ Key Laboratory of Maternal‐Fetal Medicine of Liaoning Province and Key Laboratory of Obstetrics and Gynecology of Higher Education of Liaoning Province Shenyang China; ^3^ Department of Gynecology Shandong Provincial Hospital Affiliated to Shandong First Medical University Jinan China

**Keywords:** bioinformatics, mitochondrial ribosomal protein, MRPL15, ovarian cancer, prognosis

## Abstract

**Purpose:**

To analyze the role of six human epididymis protein 4 (HE4)‐related mitochondrial ribosomal proteins (MRPs) in ovarian cancer and selected MRPL15, which is most closely related to the tumorigenesis and prognosis of ovarian cancer, for further analyses.

**Methods:**

Using STRING database and MCODE plugin in Cytoscape, six MRPs were identified among genes that are upregulated in response to HE4 overexpression in epithelial ovarian cancer cells. The Cancer Genome Atlas (TCGA) ovarian cancer, GTEX, Oncomine, and TISIDB were used to analyze the expression of the six MRPs. The prognostic impact and genetic variation of these six MRPs in ovarian cancer were evaluated using Kaplan‐Meier Plotter and cBioPortal, respectively. MRPL15 was selected for immunohistochemistry and GEO verification. TCGA ovarian cancer data, gene set enrichment analysis, and Enrichr were used to explore the mechanism of MRPL15 in ovarian cancer. Finally, the relationship between MRPL15 expression and immune subtype, tumor‐infiltrating lymphocytes, and immune regulatory factors was analyzed using TCGA ovarian cancer data and TISIDB.

**Results:**

Six MRPs (MRPL10, MRPL15, MRPL36, MRPL39, MRPS16, and MRPS31) related to HE4 in ovarian cancer were selected. MRPL15 was highly expressed and amplified in ovarian cancer and was related to the poor prognosis of patients. Mechanism analysis indicated that MRPL15 plays a role in ovarian cancer through pathways such as the cell cycle, DNA repair, and mTOR 1 signaling. High expression of MRPL15 in ovarian cancer may be associated with its amplification and hypomethylation. Additionally, MRPL15 showed the lowest expression in C3 ovarian cancer and was correlated with proliferation of CD8^+^ T cells and dendritic cells as well as TGFβR1 and IDO1 expression.

**Conclusion:**

MRPL15 may be a prognostic indicator and therapeutic target for ovarian cancer. Because of its close correlation with HE4, this study provides insights into the mechanism of HE4 in ovarian cancer.

## Graphical abstract

MRPL15 is highly expressed in epithelial ovarian cancer and is associated with poor overall survival of patients.

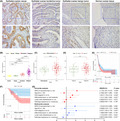

## 1 BACKGROUND

Ovarian cancer is the seventh most common cancer and eighth most common cause of death due to cancer among women worldwide.^1^ Early diagnosis can enable appropriate treatment and is the main factor associated with improving the survival rate of patients with ovarian cancer. However, because of the lack of early typical symptoms and accurate diagnostic methods, 70% of patients with ovarian cancer are already at an advanced stage when they are diagnosed,^2^ with their 5‐year survival rate rapidly declining from 92% to 29%.^3^ Surgery and chemotherapy are the main treatments for ovarian cancer. Clinically, approximately 80% of high‐grade serous ovarian cancer and high‐grade endometrioid ovarian cancer cases are sensitive to chemotherapy. However, only around 20% of patients experience no recurrence after initial treatment, whereas most patients show chronic recurrence, with 20%–30% relapsing or progressing within six months after completing chemotherapy.^4^ In women with advanced ovarian cancer, around 75% of those who relapse are incurable.^3^ Therefore, exploring the development mechanism of ovarian cancer, searching for more sensitive biomarkers, and identifying new therapeutic targets have become research trends in ovarian cancer.

Human epididymis protein 4 (HE4), which is encoded by the WAP four‐disulfide core domain 2 gene, was approved by the US Food and Drug Administration in 2008 for the early diagnosis, efficacy evaluation, and relapse monitoring of patients with epithelial ovarian cancer. HE4 is widely used in clinics for ovarian cancer diagnosis. Compared to the detection of cancer antigen 125 alone, detection of HE4 or combined detection of HE4 and cancer antigen 125 is more sensitive and specific for early diagnosis,^5^ differential diagnosis of pelvic tumors,^6^ postoperative recurrence,^7^ and prognosis judgment.^8^ Several studies have shown that HE4 promotes the proliferation, invasion, metastasis, and drug resistance in ovarian cancer.^9^ However, the specific mechanism of HE4 in ovarian cancer remains unclear, and some controversy exists. In a previous study, we used human whole‐genome microarray technology to screen complete differentially expressed genes (DEGs) after overexpression and silencing of HE4 in the epithelial ovarian cancer cell line ES‐2. In total, 717 upregulated DEGs were found by comparing HE4‐overexpressing cells and HE4‐mock cells.^10^ Deep mining of these DEGs and comprehensive data analysis are expected to provide new ideas and potential applications for studying the mechanism of ovarian cancer development and HE4‐interacting proteins.

The combination of bioinformatics and medicine is the main trend at present, and it plays an important role in the screening of biomarkers, the construction of protein‐protein interaction networks, the enrichment of pathways, and the construction of predictive models.^11^ The application of bioinformatics tools, algorithms and databases allows researchers to quickly obtain and analyze a variety of data (including genomics, transcriptomics, proteomics, and clinical data, etc.) from multiple patients in large databases.^12^ Compared with traditional biological experiments, it has the advantages of faster, more comprehensive and more cost‐effective. In recent years, the use of bioinformatics methods to identify and predict cancer biomarkers has gradually emerged, providing a large number of candidate targets for subsequent biological experimental verification, which will help promote the development of cancer precision medicine.^13^, ^14^

**FIGURE 1:**
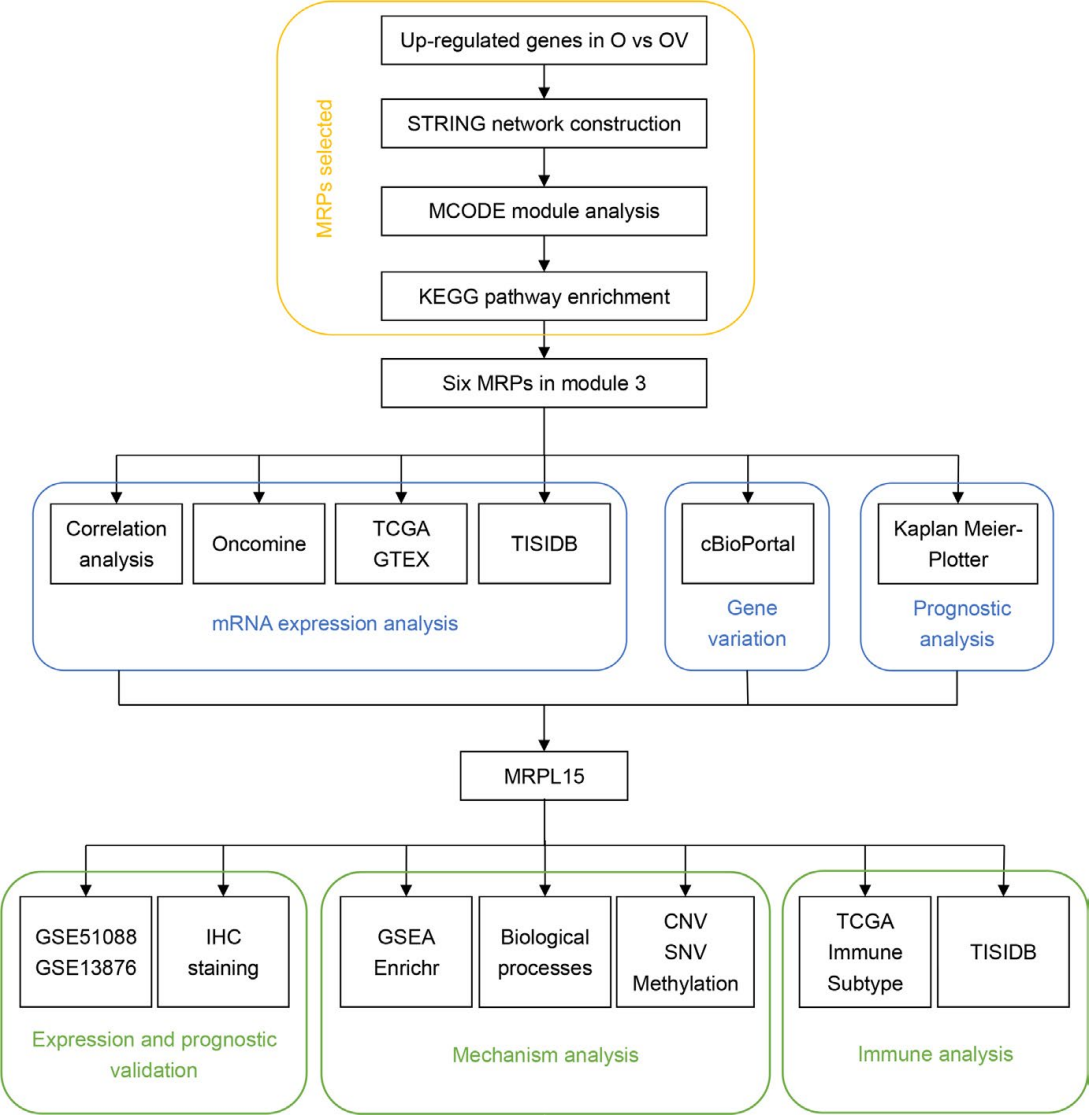
Flowchart presenting the analysis of six HE4‐related MRPs in ovarian cancer. First, as shown in the yellow box, through STRING, MCODE, and KEGG enrichment analysis of HE4‐related genes, we screened six MRPs. Second, as shown in the blue box, we performed a comprehensive analysis of mRNA expression, gene variation, and prognosis for these six MRPs. Finally, as shown in the green box, we chose MRPL15 for further verification and mechanism analysis. CNV, copy number variation; GSEA, Gene Set Enrichment Analysis; GTEX, Genotype‐Tissue Expression Portal; HE4, human epididymis protein 4; KEGG, Kyoto Encyclopedia of Genes and Genomes; O, HE4‐High; OV, HE4‐High‐vector; SNV, single‐nucleotide variants; TCGA, The Cancer Genome Atlas

Therefore, in this study, via bioinformatics analysis, we examined the role of HE4‐related proteins in promoting the malignant biological behavior of ovarian cancer and identified new candidate biomarkers and potential targets for the early diagnosis and therapy of ovarian cancer. As shown in Figure 1, we used the STRING online tool and MCODE plugin in Cytoscape to construct an interaction network of 717 upregulated DEGs and analyze the key modules in this network diagram, revealing six mitochondrial ribosomal proteins (MRPs). After analyzing the expression and prognostic impact of these MRPs in ovarian cancer, we selected *MRPL15*, which is the gene most closely correlated with the tumorigenesis and prognosis of ovarian cancer, for further verification and mechanism analysis.

## 2 MATERIAL AND METHODS

### 2.1 Protein–protein interaction network construction and key module acquisition

STRING (http://string‐db.org/) is an online database that integrates multichannel‐determined and ‐predicted protein–protein interaction (PPI) data to reevaluate all functional interactions between proteins.^15^ Cytoscape (v.3.7.2) and its MCODE plugin can be used to visualize and identify the core module of large network diagrams.^16^ We used STRING to construct an interaction network of 717 upregulated genes related to HE4 and then used the MCODE plugin to detect the closely connected region of the network.

### 2.2 Data processing

From the UCSC Xena (https://xenabrowser.net) database, we obtained gene expression data (RNA‐seq HTSeq‐Counts, *n* = 379), survival data (*n* = 731), phenotype data (*n* = 758), and somatic mutation data (VarScan2, *n* = 436) of The Cancer Genome Atlas (TCGA) ovarian cancer dataset. In addition, gene expression data from TCGA ovarian cancer dataset (RNA‐seq HTSeq‐FPKM, *n* = 379) and Genotype‐Tissue Expression Portal (GTEX) dataset (RNA‐seq TOIL RSEM FPKM, *n* = 88) were downloaded and normalized to compare the expression between ovarian cancer and normal samples. Immune subtype data were obtained from TCGA pan‐cancer dataset, and data on ovarian cancer samples were extracted for further analysis.

We selected the expression profiles GSE51088 ^17^ and GSE13876 ^18^ from GEO database (https://www.ncbi.nlm.nih.gov/geo/) to verify the expression and prognostic impact of *MRPL15* in ovarian cancer. GSE51088, which is based on the GPL7264 platform, includes 140 epithelial ovarian cancer samples, 12 ovarian borderline tumor samples, 5 ovarian benign tumor samples, and 15 normal ovarian tissue samples. GSE13876, which is based on the GPL7759 platform, includes 157 serous ovarian cancer samples from patients at an advanced stage.

To explore the relationship between MRPL15 expression with gene‐level copy number variation and DNA methylation, two studies were downloaded from the cBioPortal online database: TCGA Ovarian Serous Cystadenocarcinoma (Firehose Legacy, *n* = 606) and TCGA Ovarian Serous Cystadenocarcinoma (Nature 2011, *n* = 489).^19^ Additionally, MRPL15 expression‐related genes were obtained from cBioPortal, and 791 genes with an average Spearman's correlation coefficient of 0.3 were screened for subsequent enrichment analysis.

### 2.3 Oncomine

Oncomine (http://www.oncomine.org) is a tumor microarray database that integrates transcriptome data from multiple cancers.^20^ We compared the mRNA expression levels of MRPs in different types of malignant and normal tissues. The search thresholds were set as fold‐change =2, *p*‐value = 0.01, and gene rank =top 10%.

### 2.4 TISIDB

TISIDB (http://cis.hku.hk/TISIDB) is an online database for analyzing interactions between tumors and the immune system. This database contains 5 data sources, including 4176 records in the PubMed literature database, 8 genome‐wide high‐throughput screening datasets, genome‐wide atlas data of all patients with cancer administered immunotherapy, multiple groups data based on TCGA database, and annotation information from 7 public databases.^21^ We used TISIDB to analyze the expression levels of MRPs in patients with ovarian cancer at different clinical stages, as well as the relationship between MRPL15 and tumor immune infiltration.

### 2.5 cBioPortal analysis

cBio Cancer Genomics Portal (http://www.cbioportal.org), including data from more than 5000 tumor samples of 20 studies, is mainly used to interactively analyze multidimensional genomics data for cancer.^22^ In this study, we used cBioPortal to analyze the types and ratios of variations in genes encoding MRPs in ovarian cancer.

### 2.6 Kaplan–Meier Plotter analysis

Kaplan–Meier Plotter (http://kmplot.com) is an online platform useful for evaluating the prognostic value of gene expression in various types of cancer. It includes 22,277 gene expression data points and survival information on 1287 patients with ovarian cancer.^23^ We used this site to access the impact of some members of the MRP family on the overall survival (OS) and progression‐free survival (PFS) of patients with ovarian cancer. We compared the prognostic value of the high‐ and low‐expression groups according to the hazard ratio (HR), 95% confidence interval (CI), and log‐rank *p*‐value (*p*‐values <0.05 were considered to have a significant difference).

### 2.7 Functional and pathway enrichment analyses

Metascape (https://metascape.org/), Database for Annotation, Visualization, and Integrated Discovery (DAVID; https://davi.ncifcrf.gov), Gene set enrichment analysis (GSEA), and Enrichr (https://maayanlab.cloud/Enrichr/) were used for functional and pathway enrichment analyses. Metascape is an online tool integrating more than 40 independent databases that aims to provide comprehensive resources for gene annotation, functional enrichment, and interaction analysis.^24^ DAVID is a tool used for systematically extracting biological meaning from a large gene or protein list.^25^ GSEA considers experiments with genome‐wide expression profiles from samples belonging to two classes.^26^ Enrichr is an online annotation tool that contains 180,184 annotated gene sets from 102 gene set libraries.^27^

Metascape was used to perform KEGG pathway enrichment analysis of genes in the top six modules. The following criteria in Metascape were used to obtain a significant difference: a *p*‐value of <0.01, minimum count of 3, and enrichment factor of >1.5. KEGG pathway enrichment analysis of the top 150 mutated genes in the high‐MRPL15‐expression group was performed using DAVID and visualized using R language.

To gain insight into the mechanism of MRPL15 in ovarian cancer, GSEA and Enrichr were used. GSEA was performed using the GSEA software (v.4.0.3), and the result was visualized using the R language. In total, 379 ovarian cancer samples obtained from TCGA database were grouped into two depending on the median expression of MRPL15. H.all.v7.1.symbols.gmt of the Molecular Signatures Database (MSigDB) was downloaded and used as the reference gene set to explore the potential hallmark between the two groups. Gene set permutations were set to 1000. A nominal *p*‐value of <0.05 and false discovery rate *q*‐value of <0.25 were considered as significant. KEGG, Reactome, and BioCarta pathway enrichment analyses were performed to MRPL15‐related genes in ovarian cancer using the Enrichr online database.

### 2.8 Sample sources and clinical data

Paraffin samples and clinical data of patients with epithelial ovarian tumors admitted from December 2008 to November 2019 were collected. All samples were histopathologically diagnosed as ovarian tumors, and no patients had been administered any treatment (such as chemotherapy, radiotherapy, or hormone therapy) before surgery. In total, 118 samples, containing 81 cases of epithelial ovarian cancer, 15 cases of epithelial borderline ovarian tumor, 12 cases of epithelial benign ovarian tumor, and 10 cases of normal ovarian tissues, were included. The median ages in these four groups were 52 (19–79 years), 46 (28–71 years), 47 (28–66 years), and 43 years (32–62 years), respectively, with no significant differences between age groups (*p* > 0.05). Among the 81 patients with malignant tumors, the numbers of samples with good/moderate and poor differentiation were 37 and 44, respectively. According to the International Federation of Gynecology and Obstetrics (FIGO) 2009, 38 patients were in stage I/II and 43 patients were in stage III/IV. In addition, the number of samples with pelvic lymph node metastasis, without lymph node metastasis, and without lymph node detection were 20, 52, and 9, respectively.

### 2.9 Immunohistochemical (IHC) assay

The paraffin block of ovarian tissues was prepared in 5‐μm‐thick sections. The streptavidin peroxidase method was performed to detect the expression of MRPL15. Polyclonal antibodies against MRPL15 were purchased from Altas Antibodies (Stockholm, Sweden) and used at a dilution ratio of 1:150. Positive and negative controls were set for each batch of sections, and parallel analysis was conducted. The positive control was a slice of rectal tissue showing MRPL15 expression, and the negative control was a slice of ovarian tissue stained with phosphate buffer rather than with antibody. Staining of the cytoplasm with brown‐yellow particles was judged as MRPL15‐positive. Scores of 0, 1, 2, and 3 represented no stain, light‐yellow stain, brown‐yellow stain, and dark‐brown stain, respectively. The average percentage of positive cells in five randomly selected high‐power microscope fields was calculated and divided into five levels: <5%, 5%–25%, 26%–50%, 51%–75%, and >75%, which were respectively counted as 0, 1, 2, 3, and 4 points. Finally, the two abovementioned scores were multiplied, and the final score was determined comprehensively: 0–2 points (negative, −), 3–4 points (weak positive, +), 5–8 points (positive, ++), and 9–12 points (strongly positive, +++). Among these, 3–12 and 5–12 points were defined as positive and strongly positive expression, respectively. To eliminate scoring error, each tissue section was reviewed independently by two researchers.

### 2.10 Statistical analysis

SPSS Statistics (v.22.0; SPSS, Inc., Chicago, IL, USA) and R language (v.3.6.1) were used to statistically analyze the data, chi‐squared test or Fisher's exact probability test was used to process the count data, and *t*‐test was used to process the measurement data using the “ggpubr” R package. Kaplan–Meier analysis and log‐rank test were used for survival analysis using the “survival” and “survminer” R packages. A Cox regression model was used to explore the expression of MRPL15 and clinical pathological index via univariate and multivariate analysis. A *p*‐value of <0.05 was considered as significant. GraphPad Prism v.7.0 (GraphPad, Inc., San Diego, CA, USA) and R package, including “corrplot,” “ggplot2,” “ggstatsplot,” “plotly,” “reshape2,” “ComplexHeatmap,” and “circlize”, were used to visualize the analysis results.

## 3 RESULTS

### 3.1 Hub gene exploration in upregulated DEGs in response to HE4 in epithelial ovarian cancer cells

**FIGURE 2:**
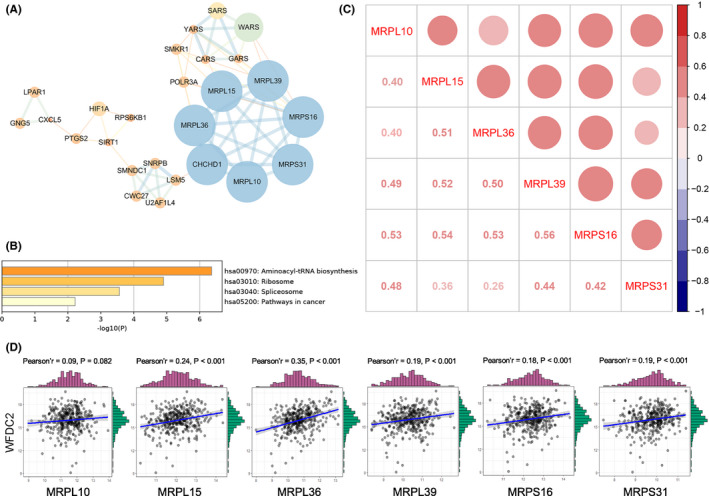
Overview of module 3 and correlation analysis between the six MRPs and their relationship with HE4. (A) PPI network of module 3 (STRING and Cytoscape). Label size and color: MCODE score in the PPI network; width and color of edges: combined score between nodes. (B) KEGG pathway enrichment analysis of module 3 using Metascape. (C) Pearson's correlation between the six MRPs in ovarian cancer in TCGA‐OV database. Red bubble: positive correlation; bubble size and color intensity: Pearson's correlation coefficient value. (D) Correlation between HE4 and the six MRPs in TCGA‐OV database. MRPs, mitochondrial ribosomal proteins; HE4, human epididymis protein 4; PPI, protein–protein interaction; KEGG, Kyoto Encyclopedia of Genes and Genomes; TCGA, The Cancer Genome Atlas; OV, ovarian cancer

STRING was used to construct the PPI network of 717 HE4‐related upregulated genes in ovarian cancer cells; 19 densely connected modules were obtained using the MCODE plugin. Table S1 shows an overview of these 19 modules. The genes in each module are listed in Table S2 according to the MCODE score. We performed visualization and KEGG enrichment analysis of the first 6 modules with MCODE scores greater than 3.00. As shown in Figure S1, modules 2, 3, 5, and 6 were significantly enriched in some cancer‐related pathways. Among the genes in these four modules, there were six members of the MRP family (i.e., MRPL10, MRPL15, MRPL36, MRPL39, MRPS16, and MRPS31) in the core part of module 3 (Figure 2A). Numerous studies have examined the role of this gene family in cancer, but analysis of these six genes in ovarian cancer remains insufficient.^28^ Therefore, we included these six genes in subsequent analysis.

As shown in Figure 2B, the enrichment results indicated that genes in module 3 were mainly involved in aminoacyl‐tRNA biosynthesis, ribosome, spliceosome, and pathways in cancer. Next, we explored Pearson's correlations between these six MRP family members and their relationship with HE4 using gene expression data from TCGA ovarian cancer dataset. As shown in Figure 2C, the expression of all six MRPs in ovarian cancer was positively correlated, with the strongest correlation being between MRPS16 and MRPL39 (*r* = 0.56, *p *< 2.2e‐16). As shown in Figure 2D, all MRPs except for MRPL10 were positively correlated with HE4, among which MRPL36 (*r* = 0.35, *p* < 0.001) and MRPL15 (*r* = 0.24, *p* < 0.001) exhibited the strongest correlation with HE4. The results for these six MRPs in our previous human whole‐genome microarrays in the human epithelial ovarian cancer cell line ES‐2 following overexpression and silencing of HE4 are shown in Table 1. These MRPs were significantly upregulated in response to HE4, with a log2 (fold‐change) greater than 1.00. This result is supported by our analysis of TCGA.

**TABLE 1:** Six MRPs in response to HE4 in ovarian cancer cells

Gene Symbol	RefSeq	O versus OV	
		Log2 (Fold Change)	*p*‐value
MRPL10	NM_148887.2	1.065127	2.75E‐02
MRPL15	NM_014175.3	1.003717	7.03E‐09
MRPL36	NM_032479.3	1.409939	2.81E‐06
MRPL39	NM_080794.3	1.439083	1.34E‐05
MRPS16	NM_016065.3	1.319883	4.62E‐06
MRPS31	NM_005830.3	1.733649	1.79E‐13

Abbreviations: HE4, human epididymis protein 4; O, HE4‐H; OV, HE4‐H‐vector.

### 3.2 mRNA expression of the six MRPs in ovarian cancer

#### 3.2.1 Differential expression levels of the six MRPs between malignant and normal ovarian tissues

**FIGURE 3:**
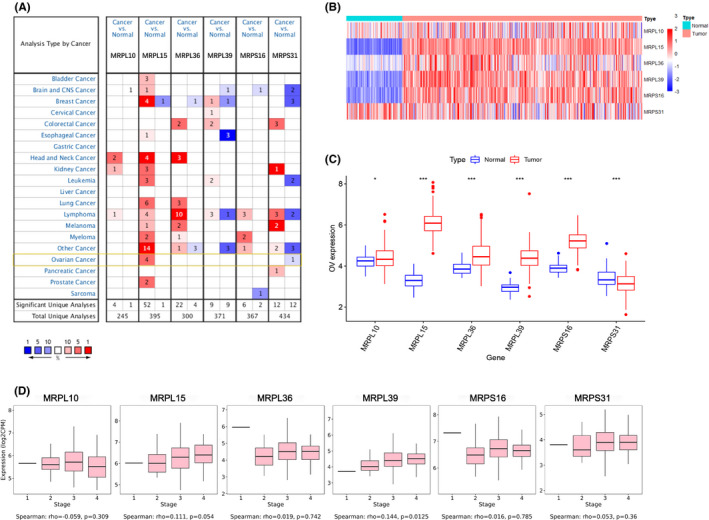
mRNA expression levels of the six MRPs in ovarian cancer. (A) mRNA expression levels of the six MRPs in different types of tumor (Oncomine). Red and blue cells represent increased and decreased gene expression, respectively. Color intensity represents the significance level of the gene expression difference. Numbers in the cells represent the number of datasets meeting the threshold. (B–C) Heatmap and boxplot of mRNA expression difference of the six MRPs between malignant and normal ovarian tissues (TCGA‐OV and GTEX). (C) MRP expression tendency in ovarian cancer at different clinical stages (TISIDB). MRPs, mitochondrial ribosomal proteins, TCGA, The Cancer Genome Atlas. OV, ovarian cancer, *p < 0.05, **p < 0.01, ***p < 0.001

Oncomine was used to explore the mRNA expression levels of the six MRPs in various types of tumors. Four studies suggested that the expression of *MRPL15* was significantly increased in ovarian cancer, whereas one study concluded that the expression of *MRPS31* was significantly decreased in ovarian cancer (Table 2, Figure 3A). Using TCGA Ovarian Statistics to compare 586 cases of serous ovarian cancer with eight cases of normal ovarian tissues, *MRPL15* was found to be significantly overexpressed in ovarian cancer (*p* = 2.13e‐8, fold‐change =2.485). Yoshihara et al. analyzed 43 cases of serous ovarian cancer and 10 cases of peritoneal tissue and concluded that *MRPL15* is overexpressed in ovarian serous cancer (*p* = 1.42e‐9, fold‐change =2.444).^28^ Moreover, Lu et al. found that compared with normal ovarian surface epithelium, *MRPL15* is significantly overexpressed in ovarian endometrioid carcinoma (*p* = 6.14e‐4, fold‐change =2.334) and ovarian serous carcinoma (*p* = 2.13e‐4, fold‐change =2.620).^29^ In addition, after analyzing 10 epithelial ovarian surfaces and 185 cases of ovarian cancer, Bonome et al. concluded that *MRPS31* is significantly downregulated in ovarian cancer (*p* = 5.47e‐6, fold‐change =−2.069).^30^ However, no significant difference was observed in Oncomine in the expression of the four remaining MRPs between malignant and normal ovarian tissues.

**TABLE 2:** Datasets of mitochondrial ribosomal proteins (MRPs) in ovarian cancer (Oncomine)

Gene	Cancer (cases)	Normal (cases)	Fold change	*t*‐Test	*p*‐value	Dataset
MRPL15	Ovarian Serous Cystadenocarcinoma (586)	Ovary (8)	2.485	17.331	2.13E‐08	TCGA
	Ovarian Serous Adenocarcinoma (43)	Peritoneum (10)	2.444	7.988	1.42E‐09	Yoshihara et al.^24^
	Ovarian Endometrioid Adenocarcinoma (9)	Ovarian Surface Epithelium (5)	2.334	4.327	6.14E‐04	Lu et al.^25^
	Ovarian Serous Adenocarcinoma (20)	Ovarian Surface Epithelium (5)	2.620	5.392	1.23E‐04	Lu et al.^25^
MRPS31	Ovarian Carcinoma (185)	Ovarian Surface Epithelium (10)	−2.069	−8.004	5.47E‐06	Bonome et al.^26^

Next, we further compared the mRNA expression difference of MRPs between 379 cases of ovarian cancer samples from TCGA and 88 cases of normal ovarian samples from GTEX database. As shown in (Figure 3B) and C, the expression of all six MRPs differed between cancer and normal samples. *MRPL10*, *MRPL15*, *MRPL36*, *MRPL39*, and *MRPS16* were highly expressed in ovarian cancer, whereas *MRPS31* showed the opposite tendency. In addition, *MRPL15* exhibited the highest expression in ovarian cancer.

#### 3.2.2 Correlation between mRNA expression levels of MRPs and FIGO stages of ovarian cancer

TISIDB was used to further explore the expression tendency of MRPs in ovarian cancer at different clinical stages (Figure 3C). The mRNA expression level of *MRPL39* was significantly increased in advanced clinical stages (Spearman's correlation: 0.144, *p* = 0.0125). In addition, the expression of *MRPL15* in ovarian cancer was increased in advanced stages but not significantly (Spearman's correlation: 0.111, *p* = 0.054). However, no significant correlations were found between expression of the four remaining MRPs and clinical stages in ovarian cancer.

In conclusion, analysis of the Oncomine and TCGA databases showed that MRPL15 is overexpressed in ovarian cancer, showing the most significant expression difference among the six MRPs. Notably, TISIDB showed that MRPL15 may also be associated with ovarian cancer progression.

### 3.3 Genetic variations of MRPs in ovarian cancer

**FIGURE 4:**
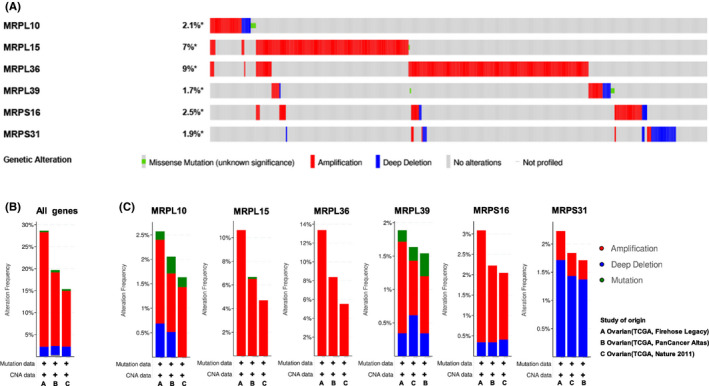
Genetic variations of MRPs in ovarian cancer (cBioPortal). (A) Summary of genetic variation rates and types of each MRP in all samples in three ovarian cancer studies. (B) Overall genetic variation rates and types of all six MRPs in each study. (C) Genetic variation of each MRP in three different studies. MRPs, mitochondrial ribosomal proteins; TCGA, The Cancer Genome Atlas

cBioPortal was used to analyze the genetic variations in the six MRPs in three studies in 1680 cases of ovarian cancer (489 cases in TCGA, Nature 2011; 585 cases in TCGA, Pan‐Cancer Atlas; and 606 cases in TCGA, Firehose Legacy). As shown in Figure 4A, the genetic variation types of these genes in all 1680 samples from the three studies were mutation, amplification, and deletion. *MRPL36* (9%) and *MRPL15* (7%) had the highest variation rates among samples in these three studies. Figure 4B presents the overall variation rates of the six MRPs in the three different studies. The total incidence rate of genetic variation in these studies was greater than 15%, with the variation rate in the study of TCGA, Firehose Legacy reaching as high as 28.64% (amplification, mutation, and deletion rates were 26.07%, 0.34%, and 2.23%, respectively).

Figure 4C shows the variation types and rates of each MRP in the three studies. In TCGA, Firehose Legacy study, *MRPL36* showed the highest incidence rate of genetic variation of 13.38% (with all cases being amplification), followed by *MRPL15*, whose amplification rate was 10.63%. In addition, other MRPs, including *MRPS16* (whose amplification and deletion rates were 2.74% and 0.34%, respectively), *MRPL10* (whose amplification, mutation, and deletion rates were 1.72%, 0.17%, and 0.69%, respectively), and *MRPL39* (whose amplification, mutation, and deletion rates were 1.37%, 0.17%, and 0.34%, respectively), exhibited high amplification rates. Hence, except for *MRPS31* (whose amplification and deletion rates were 0.51% and 1.72%, respectively), amplification is the type of genetic variation with the highest incidence rate in these ovarian cancer‐related genes.

### 3.4 Effects of MRPs on prognosis of patients with ovarian cancer

**FIGURE 5:**
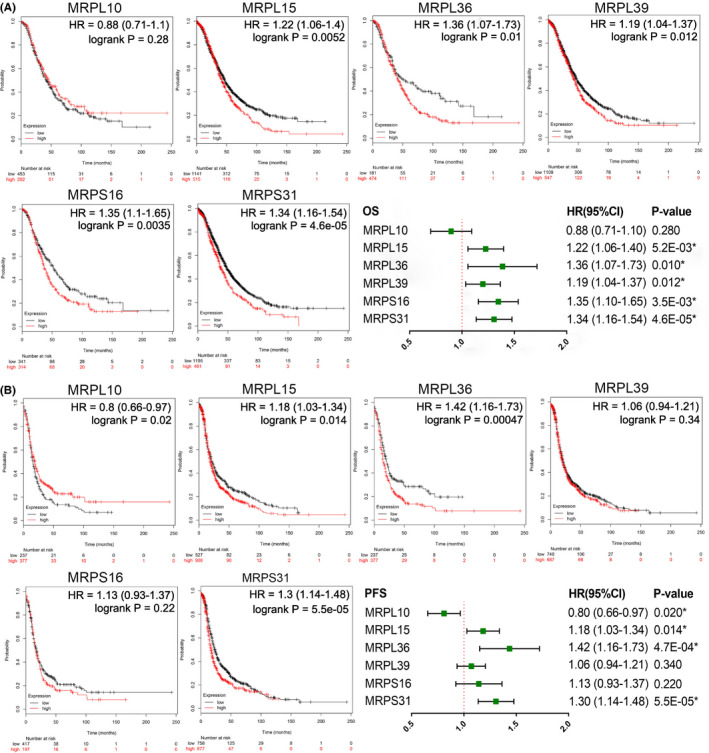
Effects of the six MRPs on the survival of patients with ovarian cancer (Kaplan Meier‐Plotter). (A) Effects of each MRP on OS shown as a forest map. (B) Effects of each MRP on the PFS shown as a forest map. CI, confidence interval; HR, hazard ratio; MRPs, mitochondrial ribosomal proteins; OS, overall survival; PFS, progression‐free survival. ^*^ *p* < 0.05

Kaplan–Meier Plotter was used to analyze the effects of the mRNA expression levels of the six MRPs on the OS and PFS of patients with ovarian cancer (Figure 5). The results suggest that overexpression of *MRPL15*, *MRPL36*, *MRPL39*, *MRPS16*, and *MRPS31* is significantly associated with poor OS (Figure 5A). Moreover, overexpression of *MRPL15*, *MRPL36*, and *MRPS31* is significantly correlated with poor PFS, whereas overexpression of *MRPL10* is correlated with better PFS (Figure 5B). Comparison of the relationship between these genes and the prognosis of ovarian cancer showed that overexpression of *MRPL15*, *MRPL36*, and *MRPS31* may lead to poor OS and PFS in patients with ovarian cancer, among which *MRPL36* showed the greatest prognostic significance.

### 3.5 MRPL15‐related signaling pathway

**FIGURE 6:**
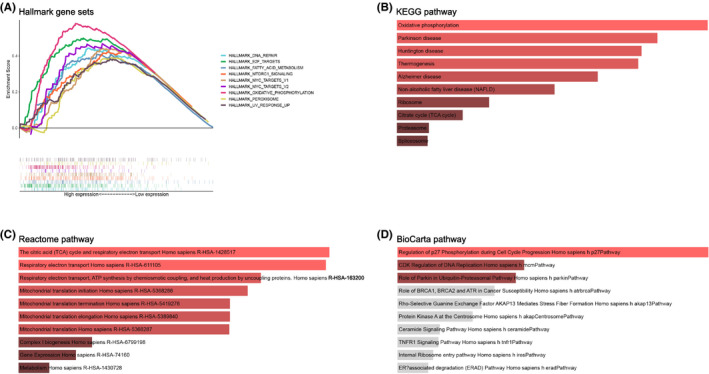
Pathway enrichment analysis of MRPL15 in ovarian cancer. (A) Hallmark gene set enrichment pathways of high‐MRPL15‐expression group in TCGA ovarian cancer database. (B) KEGG pathways enriched by MRPL15‐related genes in ovarian cancer. (C) Pathways enriched by MRPL15‐related genes in the Reactome database. (D) Pathways enriched by MRPL15‐related genes in the BioCarta database. TCGA, The Cancer Genome Atlas, KEGG, Kyoto Encyclopedia of Genes and Genomes

Only MRPL15 was highly expressed in ovarian cancer in both Oncomine analyses and the combined analyses of TCGA and GTEX. In addition, MRPL15 had a high variation rate and was significantly related to the OS and PFS of patients with ovarian cancer. To further analyze the potential function of MRPL15 in ovarian cancer, GSEA was performed between high‐ and low‐MRPL15‐expression groups in TCGA ovarian cancer database. Table 3 shows the top 10 gene sets enriched in the MRPL15‐high‐expression group (*n* = 190), for which the nominal *p*‐value <0.05 and FDR *q*‐value <0.25 were considered significant. Figure 6A shows the nine significant gene sets: “OXIDATIVE PHOSPHORYLATION,” “DNA REPAIR,” “FATTY ACID METABOLISM,” “MTORC1 SIGNALING,” “PEROXISOME,” “UV RESPONSE UP,” “E2F TARGETS,” “MYC TARGETS V2,” and “MYC TARGETS V1.”

**TABLE 3:** Gene sets enriched in the high‐MRPL15‐expression group

MSigDB collection	Gene set	NES	NOM *p*‐value	FDR q‐value
H. all v7.1 symbols gmt [Hallmarks]	HALLMARK OXIDATIVE PHOSPHORYLATION	2.13	0.004*	0.004
	HALLMARK DNA REPAIR	1.94	0.002*	0.054
	HALLMARK FATTY ACID METABOLISM	1.83	0.008*	0.098
	HALLMARK MTORC1 SIGNALING	1.79	0.019*	0.106
	HALLMARK PEROXISOME	1.76	0.006*	0.109
	HALLMARK UV RESPONSE UP	1.69	0.010*	0.151
	HALLMARK ADIPOGENESIS	1.68	0.051	0.132
	HALLMARK E2F TARGETS	1.66	0.047*	0.130
	HALLMARK MYC TARGETS V2	1.62	0.024*	0.151
	HALLMARK MYC TARGETS V1	1.61	0.036*	0.146

Abbreviations: FDR, false discovery rate; NES, normalized enrichment score; nominal *p*‐value, nominal *p*‐value.

* NOM *p*‐value <0.05.

To gain comprehensive insight into the mechanism of MRPL15 in ovarian cancer, we performed KEGG, BioCarta, and Reactome pathway enrichment analyses for 791 genes correlated with MRPL15 screened from the cBioPortal database. Enrichment analysis of the KEGG and Reactome pathways showed that MRPL15 mainly participates in oxidative phosphorylation, electron transport in the respiratory chain, and the citrate cycle. Additionally, BioCarta pathway analysis indicated that MRPL15 is also involved in regulation of p27 phosphorylation during cell cycle progression, CDK regulation of DNA replication, and ubiquitin proteasome pathway.

### 3.6 IHC staining and GEO database verification of MRPL15 expression in ovarian cancer

#### 3.6.1 MRPL15 is overexpressed in epithelial ovarian cancer

**FIGURE 7:**
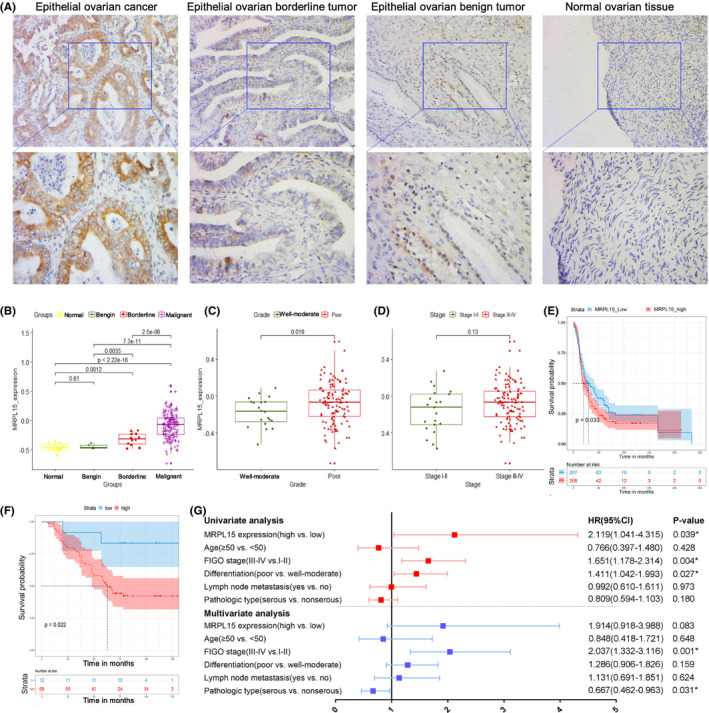
Expression of MRPL15 in epithelial ovarian tissues and its correlation with the prognosis of patients with ovarian cancer. (A) Typical immunohistochemical staining images of MRPL15 in epithelial ovarian cancer, epithelial borderline ovarian tumors, epithelial benign ovarian tumors, and normal ovarian tissues (upper: SP*200, lower: SP*400). (B–D) Expression of MRPL15 in different groups of patients and its relationship with differentiation and FIGO stages in the GSE51088 dataset. (E) Kaplan–Meier analysis of the correlation between MRPL15 expression and OS in the GSE13876 dataset. (F) Kaplan–Meier analysis of the correlation between MRPL15 expression and OS in the 81 patients with ovarian cancer. (G) Forest map of Cox regression analysis of OS in the 81 patients with ovarian cancer. ^*^ *p* < 0.05. CI, confidence interval; FIGO, International Federation of Gynecology and Obstetrics; HR, hazard ratio; OS, overall survival

IHC staining of MRPL15 was performed in 118 cases of epithelial ovarian tissues, including 81 cases of malignant tumor tissues, 15 cases of borderline tumor tissues, 12 cases of benign tumor tissues, and 10 normal tissues. As shown in Figure 7A, MRPL15 was mainly detected in the cytoplasm. The positive rate (96.30%) and high positive rate (85.19%) of MRPL15 expression in epithelial ovarian cancer tissues were significantly higher than those in epithelial borderline tumor tissues (55.33% and 40.00%, respectively), epithelial benign tumor tissues (41.67% and 16.67%, respectively), and normal ovarian tissues (30.00% and 00.00%, respectively) (all *p *< 0.05; Table 4). However, no significant difference was observed between other pairwise comparisons among epithelial borderline ovarian tumor tissues, epithelial benign ovarian tumor tissues, and normal ovarian tissues (*p* > 0.05; Table 4).

**TABLE 4:** MRPL15 expression in 118 cases of ovarian tissues

Groups	Cases	MRPL15	staining				
	(*n *= 118)	Low		High			
		(−)	(+)	(++)	(+++)	Positive rates (%)	High positive rates (%)
Normal	10	7	3	0	0	30.00	0
Benign	12	7	3	2	0	41.67	16.67
Borderline	15	7	2	1	5	53.33	40.00
Malignant	81	3	9	31	38	96.30*	85.19*

* *p* < 0.05.

#### 3.6.2 Correlation between MRPL15 expression and clinicopathologic features

Eighty‐one patients with epithelial ovarian cancer were included divided into two groups: a high‐MRPL15‐expression group (++/+++) and low‐MRPL15‐expression group (−/+). As shown in Table 5, the high positive rate of MRPL15 expression in patients with epithelial ovarian cancer at FIGO stage III/IV (93.02%) was significantly higher than in patients at FIGO stage I/II (76.32%; *p* = 0.035). However, although the high positive rate of MRPL15 expression in the poorly differentiated tumor group and lymph node metastasis group was higher than that in the well/moderately differentiated group and no lymph node metastasis group, respectively, the differences were not significant (*p* = 0.261 and 0.099, respectively; Table 5).

**TABLE 5:** Relationship between expression of MRPL15 and clinicopathological features of 81 patients with ovarian cancer

Variables	Cases	MRPL15 staining					
	(*n *= 81)	Low		High			
		(‐)	(+)	(++)	(+++)	High positive rates (%)	*p*‐value
Age							0.719
<50	30	2	3	12	13	83.33	
≥50	51	1	6	18	26	86.27	
FIGO stage							0.035*
I‐II	38	1	10	14	13	76.32	
III‐IV	43	2	1	15	25	93.02	
Differentiation							0.261
Well‐moderate	37	1	6	14	16	81.08	
Poor	44	2	3	17	22	88.64	
Lymph node metastasis							0.099
No	52	2	9	25	16	78.85	
Yes	20	1	0	3	16	95.00	
No lymphadenectomy	9	0	0	3	6	100.00	
Pathologic type							0.886
Serous	50	2	6	18	24	84.00	
Mucinous	6	0	1	1	4	83.33	
Endometrioid	7	0	1	5	1	85.71	
Clear cell carcinoma	6	0	0	1	5	100.00	
Poorly differentiated adenocarcinoma	12	1	1	6	4	83.33	

Abbreviation: FIGO, International Federation of Gynecology and Obstetrics.

* *p* < 0.05.

The GEO database was used to further validate the expression of MRPL15 and its correlation with clinicopathologic features. In GSE51088, the expression of MRPL15 in epithelial ovarian cancer was significantly higher than that in ovarian borderline tumors (*p* = 2.5e‐06), ovarian benign tumors (*p* = 7.3e‐11), and normal ovarian tissues (*p* < 2.22e‐16; Figure 7B). Furthermore, the expression of MRPL15 in ovarian borderline tumors was significantly higher than in ovarian benign tumors (*p* = 0.0035) and normal ovarian tissues (*p* = 0.0012; Figure 7B). In addition, poorly differentiated ovarian cancer samples showed higher expression of MRPL15 compared to well/moderately differentiated ovarian cancer samples (*p* = 0.019; Figure 7C). However, no significant difference was found between patients with ovarian cancer at different FIGO stages (*p* = 0.13; Figure 7D).

#### 3.6.3 Influence of MRPL15 expression on survival of patients with epithelial ovarian cancer

GSE13876, which includes 157 patients with serous ovarian cancer at an advanced stage, was selected to verify the prognostic value of MRPL15 in ovarian cancer. The results indicate that high expression of MRPL15 leads to poor OS in patients with advanced serous ovarian cancer (*p* = 0.033; Figure 7E).

In addition, the prognostic value of MRPL15 was verified in epithelial ovarian cancer by analyzing our patient samples. We followed up on 81 patients with epithelial ovarian cancer (latest follow‐up time was on November 30, 2019), among whom 18 were lost to follow‐up (22.22%). Kaplan–Meier and log‐rank tests demonstrated that the low‐MRPL15‐expression group had significantly better prognosis compared to the high‐MRPL15‐expression group (*p* = 0.022; Figure 7F). Next, Cox regression analysis was performed on these 81 cases with ovarian cancer. According to univariate Cox regression analysis, MRPL15 expression (HR = 2.119, *p* = 0.039), FIGO stage (HR = 1.651, *p* = 0.004), and differentiation (HR = 1.411, *p* = 0.027) were significantly correlated with the OS of patients with epithelial ovarian cancer. Moreover, multivariate Cox regression analysis indicated that an advanced FIGO stage (HR = 2.037, *p* = 0.001) and the pathologic type of serous ovarian cancer (HR = 0.667, *p* = 0.031) are independent risk factors for epithelial ovarian cancer (Figure 7G).

### 3.7 Molecular mechanism of MRPL15 in ovarian cancer

**FIGURE 8:**
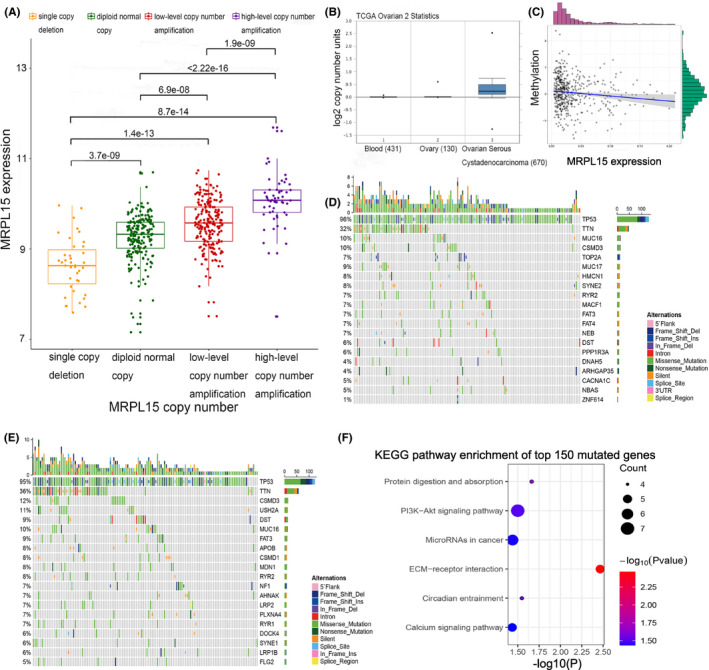
Molecular mechanisms of increased expression of MRPL15 in malignant ovarian tissues. (A) Correlation between the copy number and expression of MRPL15. (B) Difference in MRPL15 copy number between normal tissues and cancer cells in TCGA Ovarian2 Statistics (Oncomine). (C) Correlation between methylation and the expression of MRPL15. (D) Top 20 mutated genes in the low‐MRPL15‐expression group. Different types of somatic mutations are marked by different colors. (E) Top 20 mutated genes in the high‐MRPL15‐expression group. (F) KEGG pathway enrichment results of the top 150 mutated genes correlated with the overexpression of MRPL15. Bubble size: number of genes involved in a pathway; Bubble color; *p*‐value

To further explore the mechanism of overexpression of MRPL15 in ovarian cancer, we explored the relationship between MRPL15 expression and copy number variation, methylation, and somatic mutation. We analyzed the correlation between MRPL15 expression in ovarian cancer and four levels of copy number variation (single‐copy deletion, diploid normal copy, low‐level copy number amplification, and high‐level copy number amplification) using data from TCGA Ovarian Serous Cystadenocarcinoma (Firehose Legacy, *n* = 606). With an increasing copy number, MRPL15 expression was increased significantly (Figure 8A). Using Oncomine for further analysis, we found that the copy number of MRPL15 in ovarian cancer was significantly higher than that in blood and normal ovaries in TCGA Ovarian2 Statistics (Figure 8B). In addition, as shown in Figure 8C, MRPL15 expression was negatively correlated with methylation (Pearson's *r* = −0.1187, *p* = 0.0086). Therefore, high expression of MRPL15 in ovarian cancer may occur partially because of copy number variation and hypomethylation.

To explore the relationship between overexpression of MRPL15 and somatic mutations in ovarian cancer, we analyzed genes with somatic mutation in the high‐ and low‐MRPL15‐expression groups. *TP53* (96%), *TTN* (32%), *MUC16* (10%), *CSMD3* (10%), *MUC17* (9%), *HMCN1* (8%), *SYNE2* (8%), *TOP2A* (7%), *RYR2* (7%), and *MACF1* (7%) were the top 10 genes with the highest mutation rate in the low‐MRPL15‐expression group (Figure 8D), whereas *TP53* (95%), *TTN* (36%), *CSMD3* (12%), *USH2A* (11%), *MUC16* (10%), *DST* (9%), *FAT3* (9%), *APOB* (8%), *CSMD1* (8%), and *MDN1* (8%) were the top 10 genes with the highest mutation rate in the high‐MRPL15‐expression group (Figure 8E). Hence, *TP53* and *TTN* were common to both expression groups. Notably, the top 150 mutated genes in the high‐MRPL15‐expression group were collected for KEGG pathway enrichment analysis. The genes were found to be mainly involved in the activation of protein digestion and absorption, PI3 K/Akt signaling pathway, miRNA in cancer, and extracellular matrix–receptor interaction (Figure 8F).

### 3.8 Immune molecules regulated by MRPL15 in ovarian cancer

**FIGURE 9:**
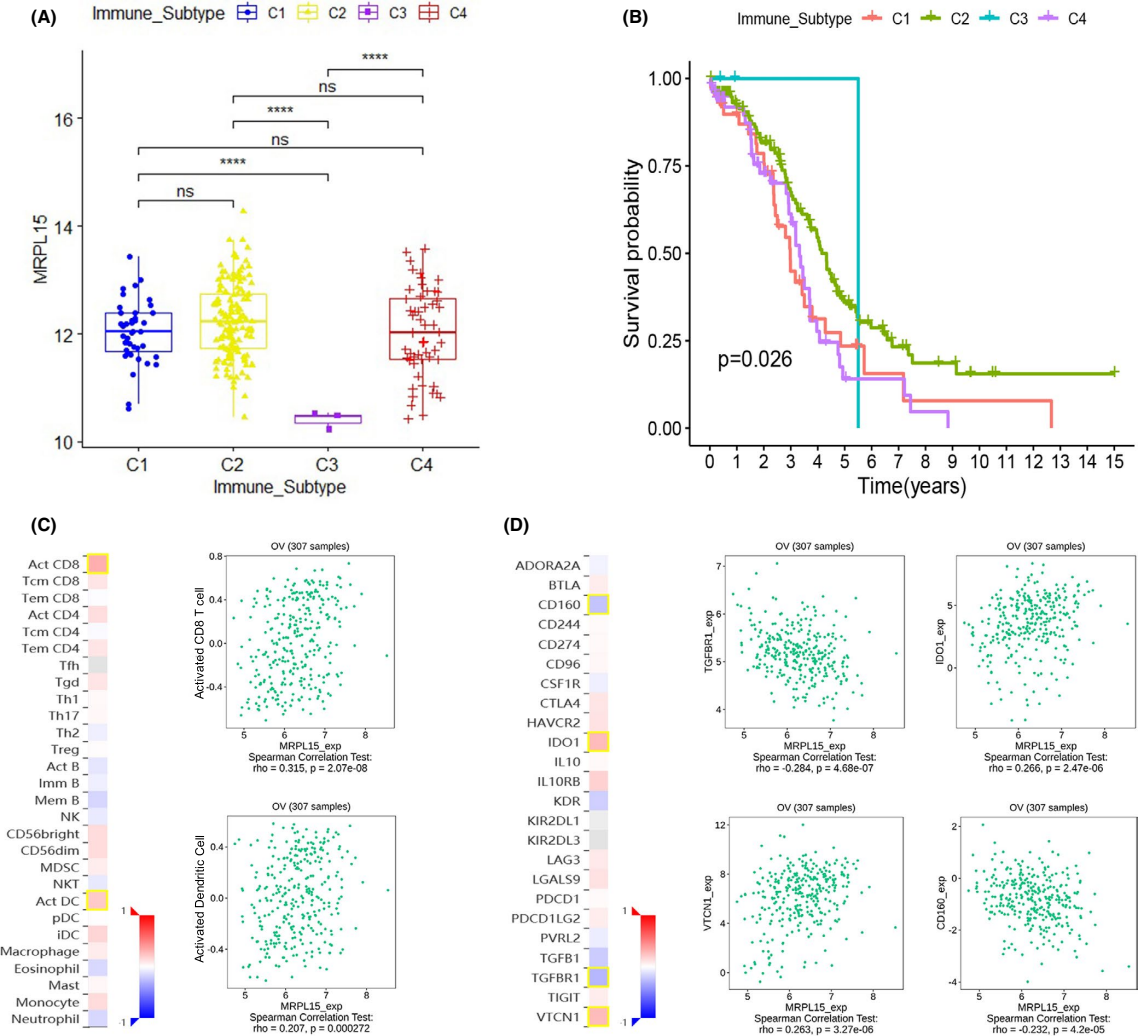
Immune analysis of MRPL15 in ovarian cancer. (A) Relationship between MRPL15 expression and immune subtype in TCGA ovarian cancer database. (B) Prognostic differences between the four immune subtypes in TCGA ovarian cancer database. (C) Correlation between MRPL15 expression and TILs (TISIDB). (D) Correlation between MRPL15 expression and immune inhibitors (TISIDB). In the heatmaps of C and D, the red and blue squares represent positive and negative correlations, respectively, and the color intensity represents the size of the Spearman correlation coefficient. The scatter plots show TILs or immune inhibitors with the strongest correlation with MRPL15 expression. TILs, tumor‐infiltrating lymphocytes

Next, we analyzed the relationship between the expression of MRPL15 and tumor immune infiltration in ovarian cancer. In TCGA ovarian cancer database, we found that MRPL15 expression was significantly related to the difference in the immune subtype of ovarian cancer, and its expression in C3 ovarian cancer was significantly lower than in the other three types (*p* < 0.0001, Figure 9A). Additionally, we analyzed the prognostic differences between these four immune subtypes of ovarian cancer in the patients. Patients with ovarian cancer with different immune subtypes exhibited significant prognostic differences (*p* = 0.026), and those with type C3 had the best survival outcomes (Figure 9B).

Using TISIDB, we analyzed the relationship between MPL15 expression, tumor‐infiltrating lymphocytes (TILs), and immune regulatory factors (immune inhibitor and immune stimulator). Figure 9C and D respectively show the first two TILs and first four immune inhibitors whose Spearman's correlation coefficient with MRPL15 expression was greater than 0.2. No strong correlation was found between MRPL15 and immune stimulators (Spearman's correlation coefficients were all <0.2). Activated CD8^+^ T cells (Spearman's rho = 0.315, *p* = 2.07e‐08) and activated dendritic cells (Spearman's rho = 0.207, *p* = 0.000272) showed the greatest correlation with MRPL15 (Figure 9C). As shown in Figure 9D, the most relevant immune inhibitors correlated with the expression of MRPL15 in ovarian cancer were TGF‐β receptor type 1 (TGFβR1; Spearman's rho = −0.284, *p* = 4.68e‐07), indoleamine 2,3‐dioxygenase 1 (IDO1; Spearman's rho =0.266, *p* = 2.47e‐06), VTCN1 (Spearman's rho =0.263, *p* = 3.27e‐06), and CD160 (Spearman's rho = −0.232, *p* = 4.2e‐05).

## 4 DISCUSSION

MRPs are the main components of mitochondrial ribosomes and are mainly involved in the translation of oxidative phosphorylation complex subunits encoded by mitochondrial DNA. Studies have shown that dysregulated expression of MRPs can cause mitochondrial translation disorders and damage to the respiratory chain, which in turn can lead to cellular metabolic disorders. In addition, some MRPs can be used as apoptosis‐inducing factors to participate in the intrinsic pathway of apoptosis, thus playing a fundamental role in regulating cell growth and apoptosis.^28^ Notably, most tumors are characterized by excessive proliferation and resistance to apoptosis. Although functional mitochondria are essential for cancer cells, the mitochondrial physiology differs between cancer and nonmalignant cells.^29^ Several studies have shown that the expression of nuclear genes encoding MRPs is altered in various types of cancer. Additionally, MRPL38 and MRPL49, among other MRPs, were confirmed to be significantly related to the invasion and prognosis of ovarian cancer.^30^, ^31^ However, studies of the role of MRPs in ovarian cancer are insufficient, and the underlying mechanism is unclear.

Our previous human whole‐genome microarray‐based study revealed that six MRPs were upregulated in response to HE4 overexpression in human ovarian cancer cells ES‐2. This is confirmed by the results of the TCGA database analysis in this research. In addition, we also found that these six MRPs play different roles in ovarian cancer. Among them, MRPL15,^32^ MRPL36,^33^ MRPL39,^31^ and MRPS31^34^, ^35^ have been confirmed by some studies to play a role in the occurrence and development of different cancers. However, our research seems to be the first to explore the relevant role of these genes in ovarian cancer.

By combining the results obtained using TCGA ovarian cancer database, GTEX database, Oncomine, cBioPortal, and Kaplan Meier‐Plotter, we found that MRPL15 plays a most significant role in ovarian cancer among the six MRPs. Further IHC and GEO validation results also showed that the expression of MRPL15 in ovarian cancer was significantly increased and was significantly related to advanced FIGO stage, poor differentiation, and poor OS in ovarian cancer patients. These results indicate that MRPL15 is not only related to the occurrence of ovarian cancer but also that it may be involved in its progression. Notably, these expression and prognosis verification results are highly consistent with our online database analysis results, indicating the potential of MRPL15 as a diagnostic and prognostic marker for ovarian cancer. Previous studies demonstrated that MRPL15 is significantly associated with poor prognosis of patients with breast cancer and that it can be used with other MRPs to establish a model to predict the prognosis of and drug efficacy for patients with estrogen receptor‐positive breast cancer.^32^ Other studies showed that MRPL15 plays an important role in maintaining the pluripotency and self‐renewal of embryonic stem cells.^36^ However, studies have not focused on MRPL15 in ovarian cancer.

To explore the mechanism of MRPL15 in ovarian cancer, GSEA, KEGG pathway, Reactome pathway, and BioCarta pathway enrichment analyses were performed. In addition to participating in basic functions related to mitochondrial oxidative phosphorylation, high expression of MRPL15 in ovarian cancer may be related to cell cycle, DNA repair, DNA replication, and the mTOR signaling pathway.

We next examined the possible mechanism of MRPL15 overexpression in ovarian cancer. The cBioPortal online database was used to analyze the genetic variation of genes encoding MRPs in ovarian cancer. The results showed that *MRPL36* and *MRPL15* had the highest genetic variation rate in ovarian cancer, with gene amplification as the most common type. DNA copy number variation includes chromosomal amplification and deletion, among which amplification is a typical genetic variation in cancer and effective acceleration mechanism to promote tumorigenesis.^37^ Many studies previously identified oncogenes in the amplified region. Analysis of the Oncomine database showed that the copy number of *MRPL15* in ovarian serous carcinoma is significantly higher than that in normal ovarian and blood samples. Further analysis of the copy number variation data in TCGA showed that with increasing gene copy number amplification, *MRPL15* expression was significantly increased in ovarian cancer (*p* < 0.05). This suggests that any increase in the *MRPL15* copy number in ovarian cancer can partly result in high expression of MRPL15. As an epigenetic mechanism, DNA methylation can change the chromatin structure to maintain the balance between transcription activation and repression. Defects in methylation may lead to the occurrence of various diseases, including cancer.^38^ Many studies have shown that methylation of certain genes in cancer is significantly reduced. We found that *MRPL15* expression in ovarian cancer was significantly negatively correlated with its methylation (Pearson's *r* = −0.1187, *p* = 0.0086), suggesting that abnormal expression of MRPL15 in ovarian cancer occurs because of the loss of methylation. However, studies are needed to confirm that hypomethylation of *MRPL15* is related to the occurrence or development of cancer.

Somatic mutations associated with MRPL15 were explored using TCGA ovarian cancer data. Compared with the low‐MRPL15‐expression group, the mutation rates of *USH2A*, *APOB*, *CSMD1*, *MDN1*, and *NF1* in the high‐MRPL15‐expression group were significantly increased. Studies have shown that the locus containing *CSMD1* is the most common in the homozygous deletion spectrum of high‐grade serous ovarian cancer, which may be a tumor suppressor gene for high‐grade serous ovarian cancer.^39^ Additionally, *NF1* is a common variant gene in high‐grade serous ovarian cancer, and its mutation is correlated to platinum‐based chemotherapy resistance in this cancer.^40^, ^41^ KEGG enrichment analysis of the top 150 mutant genes in the high‐MRPL15‐expression group showed that these genes are mainly involved in protein digestion and absorption, PI3 K/Akt signaling pathway, miRNA in cancer, extracellular matrix–receptor interaction, and other pathways. Thus, these gene mutations may be related to the high expression of MRPL15 in ovarian cancer and promote the oncogenesis and progression of ovarian cancer through the abovementioned cancer‐related pathways.

The immune response of tumors is an important process in the development and progression of tumors. After being separated from the body's immune monitoring, the tumor's malignant biological behavior is further accelerated, thereby promoting tumor proliferation, invasion, and metastasis. Although increasing evidence has demonstrated the role of the immune system in ovarian cancer, no approved immunotherapy exists for ovarian cancer for clinical use.^42^ In 2018, Vesteinn Thorsson et al.^43^ used TCGA database to classify tumors into six immune subtypes, including wound healing (C1), IFN‐γ dominant (C2), inflammatory (C3), lymphocyte depleted (C4), immunologically quiet (C5), and TGF‐β dominant (C6). There were significant differences in lymphocyte infiltration, immunoregulatory gene expression, and prognosis among different subtypes, providing new ideas for tumor immunotherapy. Our results indicate that MRPL15 expression was significantly reduced in C3 ovarian cancer, which has the best prognosis. This indicates that MRPL15 can be used as a marker for immunophenotyping of patients with ovarian cancer and for predicting the prognosis of patients. The results obtained using TISIDB indicated that MRPL15 is closely related to TILs (e.g., activated CD8^+^ T cells and activated dendritic cells) and immune inhibitors (e.g., TGFβR1, IDO1, VTCN1, and CD160). The accumulation of TILs in ovarian cancer is related to better patient prognosis. Adoptive TIL therapy is a promising immunotherapy for ovarian cancer.^38^ It has been shown that CD8^+^ T cells, natural killer cells, and dendritic cells are cytotoxic to ovarian cancer.^38^ TGFβR1,^44^ IDO1, VTCN1,^45^ and CD160^46^ are involved in tumor immune escape and are effective targets for tumor immunotherapy. Among these, IDO1 is highly expressed in most types of cancer and is associated with the poor prognosis of patients.^47^ Generally, three pathways exist downstream of IDO1 to transmit the effects of IDO1 activity^47^: activation of general control nonderepressible 2 pathway, inhibition of the mTOR pathway, and aromatic hydrocarbon receptor pathway. Interestingly, from previous enrichment analysis of the MRPL15 pathway, we predicted that MRPL15 promotes the development of ovarian cancer through the mTOR pathway. Therefore, MRPL15 may lead to immune tolerance of ovarian cancer by participating in the downstream mTOR pathway of IDO1, thereby promoting the progression of ovarian cancer. In summary, MRPL15 may play a role in the immune tolerance process of ovarian cancer by interacting with the above‐mentioned TILs and immunomodulatory molecules and may be useful as a biomarker or target for ovarian cancer immunotherapy.

## 5 CONCLUSION

In summary, MRPL15 may be a candidate biomarker and novel therapeutic target for epithelial ovarian cancer. In addition, because of its close correlation with HE4, MRPL15 may interact with HE4 to promote the oncogenesis and development of ovarian cancer. Further detailed experimental research is needed to determine the underlying roles and mechanisms of MRPs in ovarian cancer.

## CONFLICT OF INTEREST

The authors declare that they have no competing interests.

## ETHICAL APPROVAL AND INFORMED CONSENT

Ethical approval for this study was obtained from the Clinical Research Ethics Committee of Shengjing Hospital of China Medical University, and all patients provided signed informed consent in accordance with the Declaration of Helsinki.

## Supporting information

Fig S1

Table S1

Table S2

## Data Availability

TCGA data used in our study are openly available in UCSC Xena (https://xenabrowser.net) and cBioPortal (http://www.cbioportal.org). GTEX data is also downloaded in UCSC Xena (https://xenabrowser.net). In addition, GSE51088 and GSE13876 used in our study are openly available in GEO database (https://www.ncbi.nlm.nih.gov/geo/).
