# Supplementary figures and images for: MRPL15 is a novel prognostic biomarker and therapeutic target for epithelial ovarian cancer
Source: Cancer Med. 2021 May 2;10(11):3655–73. doi: 10.1002/cam4.3907 (PMC8178508; doi:10.1002/cam4.3907)

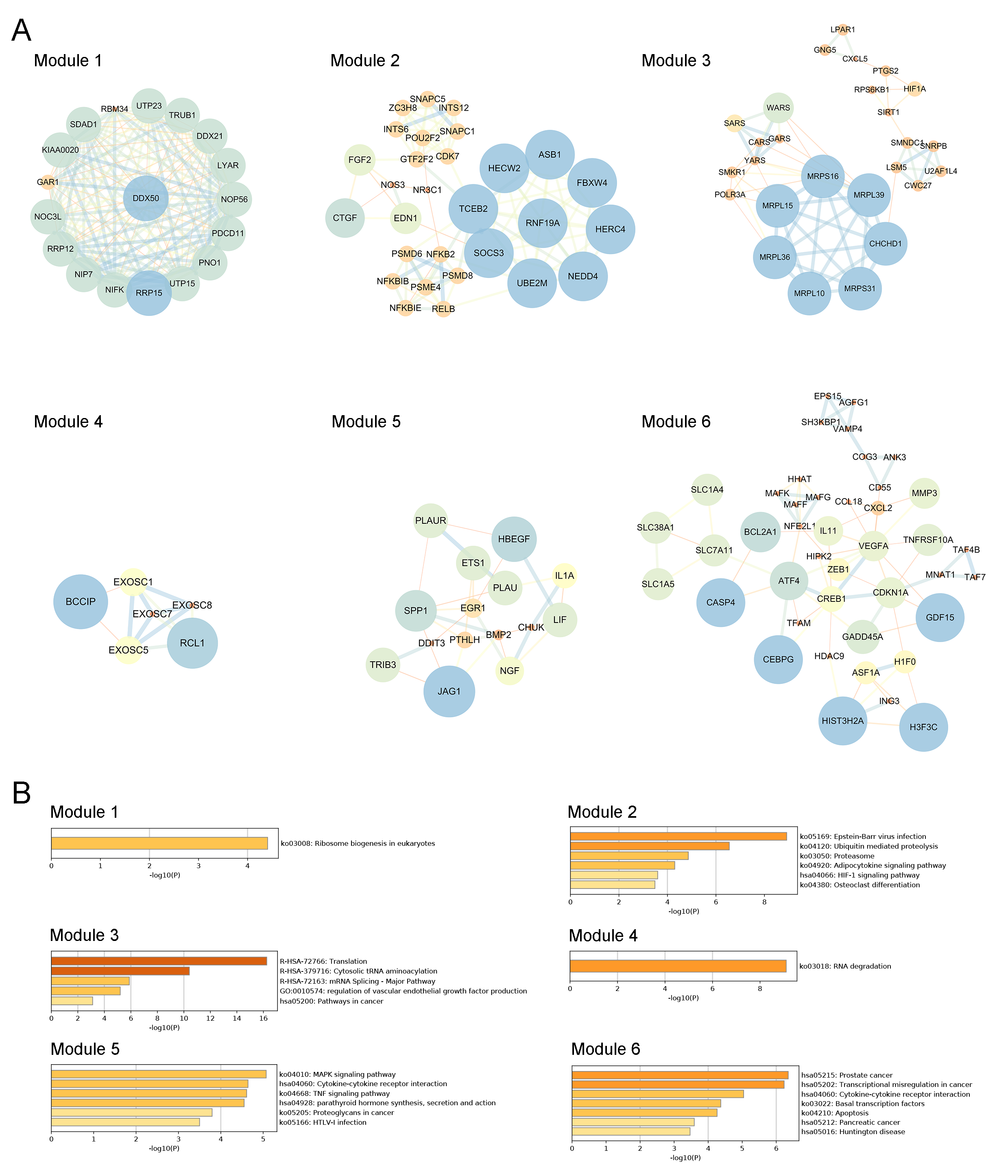

Supplement: Supplementary file 1 — Fig S1 [file CAM4-10-3655-s001.tif]
