# Supplementary material for: MRPL15 is a novel prognostic biomarker and therapeutic target for epithelial ovarian cancer
Source: Cancer Med. 2021 May 2;10(11):3655–73. doi: 10.1002/cam4.3907 (PMC8178508; doi:10.1002/cam4.3907)
Supplement: Supplementary file 2 — Table S1 [file CAM4-10-3655-s003.docx]

| MCODE Cluster | Number of nodes | Number of edges | MCODE Score |
| --- | --- | --- | --- |
| 1 | 18 | 144 | 16.941 |
| 2 | 29 | 103 | 7.357 |
| 3 | 26 | 69 | 5.520 |
| 4 | 6 | 12 | 4.800 |
| 5 | 15 | 27 | 3.857 |
| 6 | 42 | 75 | 3.659 |
| 7 | 5 | 6 | 3.000 |
| 8 | 3 | 3 | 3.000 |
| 9 | 3 | 3 | 3.000 |
| 10 | 3 | 3 | 3.000 |
| 11 | 3 | 3 | 3.000 |
| 12 | 3 | 3 | 3.000 |
| 13 | 3 | 3 | 3.000 |
| 14 | 3 | 3 | 3.000 |
| 15 | 3 | 3 | 3.000 |
| 16 | 3 | 3 | 3.000 |
| 17 | 3 | 3 | 3.000 |
| 18 | 3 | 3 | 3.000 |
| 19 | 3 | 3 | 3.000 |
